# Toxicity and Neuropharmacological Effects of Elenine

**DOI:** 10.1155/2011/312524

**Published:** 2011-05-26

**Authors:** Eduardo Navarro, S. J. Alonso, R. Navarro

**Affiliations:** ^1^Department of Pharmacology, Faculty of Medicine, University of La Laguna, La Laguna, 38071 Tenerife, Spain; ^2^Department of Medical and Surgical Sciences, University of Las Palmas, Gran Canaria, 35001 Canary Islands, Spain

## Abstract

Elenine is the aglycone of elenoside, a cytotoxic arylnaphthalene lignan (NSC 644013-W/1) derived from *Justicia hyssopifolia*. (Family: Acanthaceae). Elenoside is a *β*-D-glucoside, with a similar chemical structure to etoposide, exhibiting central depressant activity. In the present study, elenine was given to mice and rats at doses of 10, 20, and 40 mg/kg. Acute toxicity (24 h) and general behaviour in mice was studied as well as its effects on muscular relaxant activity, locomotor activity (Varimex test), and the open-field test and were compared with 10 mg/kg of chlorpromazine. Elenine produced a reduction in the permanence time in muscular relaxant activity (traction test). Spontaneous activity was lower in the Varimex test. The ambulation and rearing were lower compared with the control group, and an increase in boluses was observed in the open-field test. Thus, it can be concluded that elenine has central sedative effects at lower doses than those used with elenoside and has a possible application in conditions of anxiety.

## 1. Introduction

*Justicia hyssopifolia, *in the Canary Islands, is believed to have laxative, purgative, anticarcinogenic as well as antiviral, and insecticidal properties [1, 2]. A large number of arylnaphthalene lignans have been isolated from different species of Justicia, many of them exhibiting diverse biological activities of which the most noteworthy are their antitumoral, insecticidal, cardiotonic, analgesic, inhibitor of lipid peroxidation and anti-inflammatory properties. Activities of interest also include its action on the central nervous system. The lignans are capable of acting both as depressants and antidepressants. Thus, (+)-nortrachelogenin causes depression in rabbits [3], and prostalandins A, B and C produce a mild depression in rats and mice [4]. Bisepoxylignan glycoside simplexoside has CNS-depressant activity in mice and rats while the genin is a stimulant [5]. 

It is well known that etoposide and other podophyllotoxin derivatives, used for their antineoplastic or antimitotic effects, affect the nervous and gastrointestinal systems. Therefore, central effects are delayed in onset and prolonged after onset and include acute psychotic reactions, hallucinations, confusion, dizziness, stupor, ataxia, hypotonicity, seizures, and coma. Peripheral and autonomic neuropathies develop later and may result in paresthesia, reduced reflexes, muscle weakness, and acute dystonic reaction [6, 7]. Gastrointestinal disorders may be more common after oral administration [8].

In a previous research [9], we reported the isolation of an arylnaphthalene lignan named J_2_ and its aglycone J_1_ from *Justicia hyssopifolia* and which we called elenoside and elenine, respectively, the first as a *β*-D-glucoside. Elenoside (NSC 644013-W/1) displayed cytotoxic activity when studied by the human tumour cell line panel of the US National Cancer Institute (NCI) [10]. Elenoside is a lignan with an action similar to that of purgative and prokinetics drugs and could be an alternative to cisapride in the treatment of gastrointestinal diseases as well as a preventive therapy for the undesirable gastrointestinal effects produced by opioids used for mild to moderate pain [11]. Furthermore, elenoside showed a decrease in the parameters of general observable behaviour evaluated by behavioural, neurological, autonomic and toxic reactions [12, 13]. Elenoside showed depressant central nervous activity at doses 25 and 50 mg/Kg [14]. In the present research, general observable behaviour and the CNS-depressant activity of elenine at doses of 10, 20, and 40 mg/Kg was studied. Elenine has central sedative effects at lower doses than those used with elenoside and has a possible application in conditions of anxiety. 

## 2. Material and Methods

### 2.1. Chemicals and Treatment

**Figure 1:**
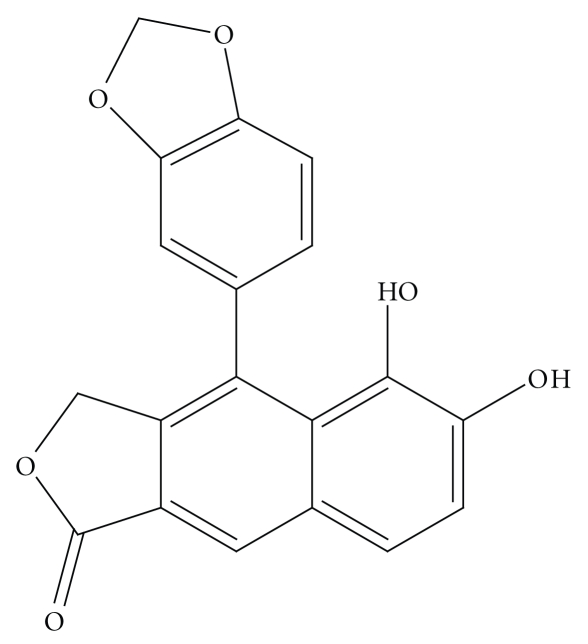
Elenine.

Elenine (3-hidroxy-metyl-1-methoxy-5,6-methylene-dioxy-4-(3,4-methylenedioxyphenyl)-2-naftoic acid lactone) (Figure 1) was obtained as described and its purity was established on the basis of the spectra (1H, 13CNMR, and mass) data [9]. 

Elenine was dissolved in a mixture of propyleneglycol-ethanol-Tween 80 (40 : 10 : 50) and administered intraperitoneally at doses of 10, 20, and 40 mg/kg. The control group only received the vehicle propylene glycol-ethanol-Tween 80 (40 : 10 : 50). Chlorpromazine hydrochloride (Rhone-Poulenc), 10 mg/kg i.p. with the same vehicle, was used. Drugs were injected into animals i.p. 1 h before testing. 

### 2.2. Animals

Swiss albino male mice (weighing 30–40 g) and Sprague-Dawley male rats (weighing 200 to 250 g) were used. The animals were housed under normal laboratory conditions at 22°C on a standard light-dark schedule (12 : 12; lights on: 0800 to 2000) and were given free access to standard laboratory diet and water. The animals were habituated to the testing apparatus, on several occasions for periods of two hours, on 2 consecutive days before real Varimex or open-field testing. Habituation was carried out in the absence of drug effects. Hypothermia in mice was observed in a subjective manner (by manual touch). This is the reason why (+) is used as the score in the Irwin test. The animals were assigned to randomised groups of 10 animals each. Animal care complied with the Guide for the Care and Use of Laboratory Animals. The study plan was approved by the local ethical committee for animal experimentation of University of La Laguna. 

### 2.3. Toxicity of Elenine and LD_50_ Determination

A total of 60 male mice (30–40 g) were allotted to the different control and test groups. The control group received the vehicle, and the test groups received doses of 150, 176, 207, 244, 287 and 338 mg/kg of elenine. The animals were kept in plastic cages (10 animals per cage), and mortality, physiological and behavioural signs of toxicity, such as motor activity, ataxia, ventral reclining, respiratory distress, hypothermia, piloerection, and palpebral closure, were noted before dosing and 0.5, 1, 2, 4, 6, 12, 24 h after dosing every day for a 7-day period [13]. The LD_50_ 24 h of elenine was calculated using the Spearman-Kärber method [15]. 

### 2.4. General Behaviour: Irwin Test

The Irwin test is a systematic observational procedure for assessing and scoring the effects of drugs on the behavioural and physiological state of rodents. The method is part of the safety pharmacology core battery recommended by International Committee for Harmonization to detect potential adverse effects of candidates on the central nervous system (CNS) before human testing. When applied at an early stage of drug development, this test is particularly suitable to (a) screen and select compounds against unwanted CNS effects, (b) understand the mechanism underling these effects, (c) help improve the structure-activity relationship, and (d) possibly, reveal novel therapeutic effects. By using an appropriate dose range for each test molecule, it is possible to obtain information on its pharmacological profile, on the intensity and the duration of its effects, and on the specificity of these effects. We used the original Irwin test with minor modifications. Mice (30–40 g) receive the test compound by intraperitoneal route and are observed at 30, 60, 120 and 180 minutes after administration. At each observation time, 25 parameters are scored using a rigorous standardized procedure based on the one described by Irwin. These parameters are distributed as follows: 9 items for the behavioural profile (body position, locomotor activity, transfer arousal, touch escape, positional passivity, toe pinch, corneal, pinna, and tail elevation); 10 items for the neurological profile (body tone, abdominal tone, limb tone, grip strength, wire manoeuvre, righting reflects, hypotonic gait, tremors, twitches, and convulsions); 6 items for the autonomic profile (palpebral closure, diarrhoea, piloerection, hypothermia, respiratory rate, and skin colour) [16].

This test was performed with fourty male mice (30–40 g) that were assigned to four randomized groups of 10 animals each. The groups were observed at 30, 60, 120, and 180 min. following the intraperitoneal administration of the vehicle, 10, 20, and 40 mg/kg of elenine. 

### 2.5. Lim Test

The measurements of the animal behaviour were also determined as described by Lim [17], with minor modifications. Three parameters are scored: muscle tone or the amount of tension or resistance to movements in a muscle, spontaneous movements or activity produced or performed through natural processes without external influence, righting reflex or the ability to assume an optimal position when there has been a departure from it. This test was performed using 40 male mice (30–40 g) that were assigned to four randomized groups of 10 animals each. The groups were observed before and for 1, 2, and 3 hours after administration of the vehicle, with 10, 20, and 40 mg/kg of elenine. The mice were evaluated for loss of spontaneous movements, loss of muscular tone and loss of righting reflex. 

### 2.6. Physiological or Behavioural Changes Following a 7-Day Daily Treatment

Fourty male mice (30–40 g) were assigned to four randomized groups of 10 animals each. The groups were observed at 30, 60, 120, 180, and 240 min following intraperitoneal administration of the vehicle, with 10, 20, and 40 mg/kg of elenine every day for a 7-day period. The animals were kept in plastic cages (10 animals per cage). Changes in neurological, autonomic, and toxic reactions and behaviour were noted. 

### 2.7. Muscular Relaxant Activity (Traction Test)

The experiment was performed using 50 male mice (30–40 g) that were assigned to four randomised groups of 10 animals each receiving the vehicle, with 10, 20, and 40 mg/kg of elenine or chlorpromazine, 10 mg/kg i.p. The forepaws of each mouse were placed in a small twisted wire rigidly supported above a bench top. Normal mice grasped the wire with their forepaws and, when they were allowed to hang free, they placed at least one hind foot on the wire within 5 seconds. Inability to place at least one hind foot was considered a failure to traction [18]. 

### 2.8. Locomotor Activity (Varimex Test)

The name Varimex suggests this is an instrument capable of measuring a variety of movements. Motor activity was measured in an activity meter (Varimex, Columbus Instruments), provided with horizontal electromagnetic sensors, which consisted of two standard plastic rat cages lined with a layer of ground corn cob bedding. When inductive horizontal sensors are used, Varimex can be used in the same way as the two alternative working activity meters which can operate either in normal mode or selective mode. 

The inductive sensor is composed of six inductive coils connected in series and tuned to resonance with an additional internal capacitor. When an animal approaches one of the coils, the voltage signal is subsequently amplified and recorded by the Varimex counter. Normal operation is characterized by a high operating frequency, of resonance circuit which results in higher sensitivity for movements of animal bodies.

In selective operation, the sensor operates at a relativity lower frequency so that the animals' body does not generate strong enough signals to be recorded. Only when an animal moves (which couples well with the magnetic field induced by the sensor) does the animal movement result in a measurable pulse. On this basis, animals marked by metal loops can be selectively monitored, while animals without the metal loops are not visible to sensor. The best sensitivity of Varimex in selective mode is obtained when the plane of the coil (loop) attached to the animal is parallel to sensor coils, that is, horizontal plane. The animals were habituated, on several occasions for periods of two hours, to the testing apparatus before real testing on 2 consecutive days. Habituation was carried out in the absence of drug effect. The following day, according to Tyler and Tessel, one animal was placed in each cage for 1 hour, but the activity was only recorded for the second half-hour. Thus, initial locomotor activity was not measured [19]. Groups of 10 male rats received vehicle, with 10, 20, and 40 mg/kg of elenine or chlorpromazine, 10 mg/kg i.p. A total of 50 male rats (200–250 g) were distributed among the different control and test groups. 

### 2.9. Open-Field Test

The open-field test has been used as an animal model of emotional behaviour. Animals with higher levels of motor activity, and lower boli rates have been taken to indicate that they exhibit less emotionality (anxiety, fearfulness). Some authors suggest that the open-field test shows aspects of many types of behaviour including exploration, locomotor activity and anxiety. The animals were habituated, on several occasions for periods of two hours, to the apparatus for open-field testing on two consecutive days before testing. Habituation was carried out in the absence of drug effect. In the open-field test, which was performed on the following day, one animal was placed in the open-field apparatus for 5 min, but the activity was only recorded for the following 5 min. 

The open-field apparatus consisted of a circular field of a 76 cm diameter and a nontransparent white wall 55 cm high. The floor was marked off into 17 sectors divided by two circles into outer, inner, and central regions. A 60 W light bulb was hung above the centre of the open field. Each rat was individually placed into an external sector of the open field. The total number of sectors entered by the animal (at least three quarters of the body), the number of rearing, and the number of defecation boluses were recorded over a test period of five minutes. The floor of the open field was cleaned with water when the animals were changed [20]. This test was performed on five groups of 10 male rats (200–250 g) each and 10, 20, and 40 mg/kg of elenine or 10 mg/kg of chlorpromazine in the vehicle were administered i.p. 

### 2.10. Measurement of Blood Pressure

Blood pressure in conscious rats was measured in an LE 5000 Digital Pressure meter. The LE 5000 is an electronic instrument designed to provide visual display and measurement of arterial pressure (systolic and diastolic), and pulse rate in animals. This is a system that is widely used to determine maximum and minimum pressure; it functions by causing an artery to collapse (sphyngomanometer), using pressure produced by cuff; blood pulses are picked up by an appropriate transducer unit. 

The overall operation of the apparatus is as follows: an internal pressure transducer with a high resolution feeds a signal to an analogical/digital converter that is equivalent to the air pressure that exists at each moment in the pneumatic line (and, therefore, the one present in the cuff). This air pressure can be produced manually with a pear, or automatically with a pressure cylinder. The analogical/digital converter activates a three-digit display that provides a readout of the value of successive pressures, expressed in millimetres of mercury and with a resolution of 1 mmHg.

Another transducer, this one external, records blood pulses and transmits this information to the control circuit which cuts off the display when it fails to receive a signal due to the collapsing of an artery, and therefore the systolic pressure (SP) is projected. When the pressure in the line drops, the display again readsout values corresponding to the pressure, until such time as the amplitude of the cardiac pulsations regains 80% of their initial value; at this time, the display is cut off again, and the value of diastolic pressure (DP) can be read. The pulse rate is also shown on the display, in beats per minute (from 1 to 999). 

Pressure in rats is recorded in the tail and in the caudal artery. It is necessary to immobilize them, using a holder, and it is convenient to subject them to a vessel dilation process beforehand, via heat treatment, so that arterial pulsations are as clearly defined as possible and free from interference from other signals which are generally due to respiration, stress or muscular tremors. 

Fifteen male rats (200–250 g) were assigned to three randomized groups of five animals each. The blood pressure was observed at 0, 30, 60, and 120 minutes following the intraperitoneal administration of 10, 20, and 40 mg/kg of elenine. 

### 2.11. Statistical Analysis

Testing out the homogeneity of variance among groups was performed using the SPSS Statistic Program. As a result, the Kruskal Wallis Test (ANOVA) followed by the Mann-Whitney test were conducted for the comparison between the mean study groups in the Varimex and Open field test using the Prism Program. The chi-square test was used for analyzing the results obtained in the Irwin and the Lim test. Differences were considered significant when associated with a probability of 5% or less. 

## 3. Results

### 3.1. Acute Toxicity

The LD_50_ (24 h) of elenine in mice was 254 mg/Kg by i.p. route. This result indirectly indicates the absence of severe toxic effects. Death was not induced in the remainder of the animals during the following 7 days. 

### 3.2. General Behaviour: Irwin Test

Elenine produced a reduction of spontaneous activity, reflected by body position and locomotor activity scores, which tend to decrease as a function of time and dose (*P* < .05 control versus 10, 20, and 40 mg/Kg elenine at 30, 60, 120, and 180 min). There was a decrease in the transfer arousal and touch escape (*P* < .05 control versus 10, 20, and 40 mg/Kg elenine at 30, 60, 120, and 180 min) and an increase of positional passivity (*P* < .05 control versus 10, 20, and 40 mg/Kg elenine at 30, 60, 120, and 180 min) representing the motor affective response. The sensorimotor response, evaluated by toe pinch (ipsilateral flexor reflex), and corneal and pinna reflex, revealed a reduction of the responses *P* < .05 control versus 10, 20, and 40 mg/Kg elenine at 30, 60, 120, and 180 min). The responses showed a reduced behavioural state.

The neurological state was evaluated by muscle tone (body, abdominal and limb tone, grip strength and wire manoeuvre), equilibrium and gait (righting reflex and hypotonic gait). Elenine produced a decrease in the muscle tone, equilibrium and hypotonic gait responses (*P* < .05 control versus 10, 20, and 40 mg/Kg elenine at 30, 60, 120, and 180 min).

Nonobserved excitation was evaluated by phenomena such as tremors, twitches and convulsions. Autonomic measurements revealed a slight palpebral closure (*P* < .05 control versus 10, 20, and 40 mg/Kg elenine at 30, 60, 120, and 180 min). Diarrhoea and a decreased respiratory rate were observed. The effects of elenine on the behavioural and physiological state (neurologic and autonomic) diminished as a function of time and administered dose (Table 1). 

### 3.3. Lim Test

Elenine was compared with a major tranquilizer (Chlorpromazine) on parameters affected by this tranquilizer, such as spontaneous movements, muscle tone and righting reflex.  Table 2 shows an increase in the number of animals with loss of spontaneous movement (*P* < .05 control versus experimental groups) and muscle tone (*P* < .05 control versus experimental groups) at 1 and 2 h, after administration of 10, 20 and 40 mg/Kg of elenine and 10 mg/Kg of chlorpromazine. No statistical differences between 2 and 3 h after administration of 10, 20 and 40 mg/Kg of elenine and 10 mg/Kg of chlorpromazine were observed. Loss of righting reflex at 1, 2, and 3 h after administration of 40 mg/kg of elenine and 10 mg/Kg of chlorpromazine was observed. 

### 3.4. Physiological or Behavioural Changes Following a 7-Day Daily Treatment

An examination of the general pharmacological effects of elenine observing changes in behaviour, neurological, autonomic parameters a 7-day daily treatment revealed that elenine, on the first day, at doses of 10, 20 and 40 mg/kg reduced spontaneous activity (body position, locomotor activity) as a function of time and dose (*P* < .05 control versus 10, 20, and 40 mg/kg elenine at 30, 60 120, and 180 min). At 240 min a normal spontaneous activity with respect to control was obtained. There was a decrease in the motor affective response (transfer arousal, touch escape, positional passivity) (*P* < .05 control versus 10, 20, and 40 mg/kg elenine at 30, 60, 120, and 180 min). At 240 min a normal score respect to control was observed. A dose-dependent reduction of sensory motor (toe pinch corneal and pinna) was also observed (*P* < .05 control versus 10, 20, and 40 mg/kg elenine at 30, 60, 120, and 180 min). Elenine produced a decrease in muscular tone (body tone, abdominal tone, limb tone and grip strength), a loss and with equilibrium and hypotonic gait (*P* < .05 control versus 10, 20, and 40 mg/kg elenine at 30, 60, 120, and 180 min). No CNS excitation (i.e., tremors, twitches and convulsions) was observed. The presence of palpebral closure, hypothermia, reduction in respiratory rate and the manifestation of diarrhoea indicates some autonomic nervous system involvement. A normal score for these effects was obtained at 240 min. These items were evaluated over the following six days and no changes in parameters (behaviour, neurological, and autonomic) were observed with respect to the preceding day. 

### 3.5. Muscular Relaxant Activity

**Figure 2:**
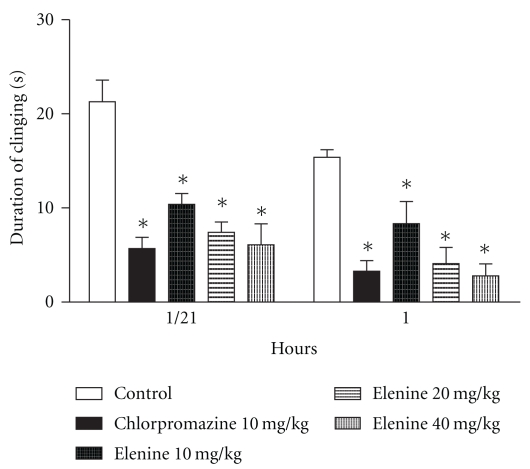
Effect of elenine 10, 20, and 40 mg/kg and chlorpromazine 10 mg/kg on muscular relaxant activity. The data represent the mean ± SEM of the permanence time (sec.) on the stainless steel wire at 30 and 60 min. *P < .05 versus control group.

Figure 2 shows the time that animals remained clinging to the stainless steel wire at the 30 and 60 min. Elenine produced a reduction in the permanence time with doses of 10 mg/kg (*P* < .05), 20 mg/kg (*P* < .05), 40 mg/kg (*P* < .05) and chlorpromazine, 10 mg/kg (*P* < .05) at 30 min. compared with the control group. Similar results were obtained at 60 min. when 10 mg/kg elenine (*P* < .05), 20 mg/kg (*P* < .05) and 40 mg/kg elenine (*P* < .05) and chlorpromazine, 10 mg/kg (*P* < .05) were compared with the control. No statistical differences between elenine (20 and 40 mg/Kg) and chlorpromazine (10 mg/Kg) at 30 and 60 min. were observed. 

### 3.6. Locomotor Activity

**Figure 3:**
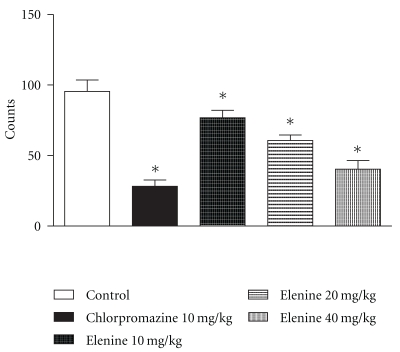
Effect of elenine 10, 20, and 40 mg/kg and chlorpromazine 10 mg/kg on locomotor activity. The data represent the mean ± SEM of number counts during 60 min. *P < .05 versus control group.

Figure 3 shows the number of counts during the first 60 min after administration of vehicle (control), elenine 10, 20 and 40 mg/kg or chlorpromazine, 10 mg/kg i.p. The spontaneous activity was lower after administration of elenine to doses of 10 mg/kg (*P* < .05), 20 mg/kg (*P* < .05), 40 mg/Kg (*P* < .05) and chlorpromazine, 10 mg/kg i.p (*P* < .05) compared with the control. Statistical differences between three doses of elenine (10, 20, and 40 mg/Kg) were observed. No statistical differences between elenine (40 mg/Kg) and chlorpromazine (10 mg/Kg) were observed. 

### 3.7. Open-Field Test

**Figure 4:**
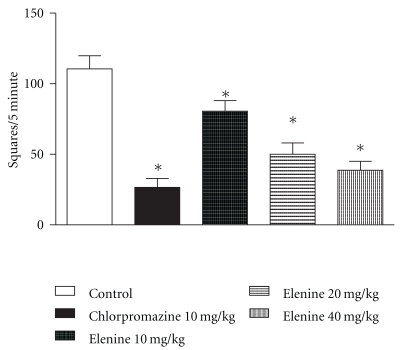
Effect of elenine 10, 20, and 40 mg/kg and chlorpromazine 10 mg/kg on open-field test: number of squares crossed for 5 min. The data represent the mean ± SEM. *P < .05 versus control group.

Figure 4 shows that, in the open-field, elenine at doses of 10, 20, and 40 mg/kg and chlorpromazine 10 mg/Kg produced a decrease in the number of squares compared with the control group (*P* < .05). No statistical differences between elenine (40 mg/Kg) and chlorpromazine 10 mg/Kg were observed. Chlorpromazine decreased the number of squares in open-field test with respect to control (*P* < .05) and doses of 10 (*P* < .05), 20 (*P* < .05) and 40 mg/kg (*P* < .05) of elenine. 

**Figure 5:**
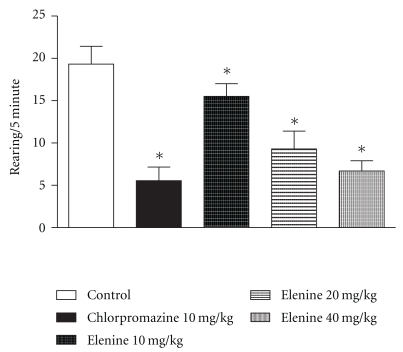
Effect of elenine 10, 20, and 40 mg/kg and chlorpromazine 10 mg/kg on open-field test: rearing patterns for 5 min. The data represent the mean ± SEM. *P < .05 versus control group.

A reduction in rearing scores (Figure 5) at doses of 10, 20, and 40 mg/kg compared with the control group was obtained and statistical differences between three doses of elenine were observed. Chlorpromazine produced a reduction in the number of rearings in the open-field test in comparison with the control group (*P* < .05), 10 and 20 mg/kg (*P* < .05) of elenine, respectively. 

**Figure 6:**
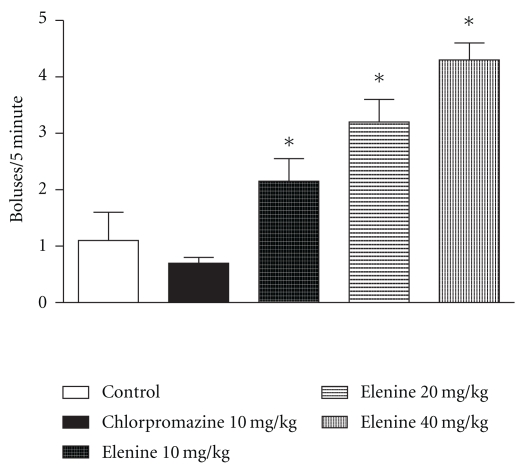
Effect of elenine 10, 20, and 40 mg/kg and chlorpromazine 10 mg/kg on open-field test: boluses for 5 min. The data represent the mean ± SEM. *P < .05 versus control group.

An increase in the number of faecal boluses (Figure 6) with 10 (*P* < .05), 20 (*P* < .05) and 40 mg/kg (*P* < .05) of elenine compared with the control group was observed. No statistical differences between the control and chlorpromazine group were observed. 

### 3.8. Measurement of Blood Pressure

**Figure 7:**
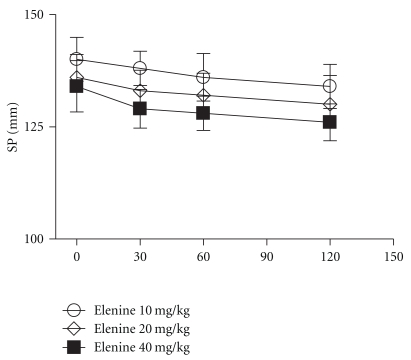
Effect of elenine 10, 20, and 40 mg/kg on Systolic Pressure (SP mm). The data represent the mean ± SEM for 5 rats at 0, 30, 60, and 120 min.

**Figure 8:**
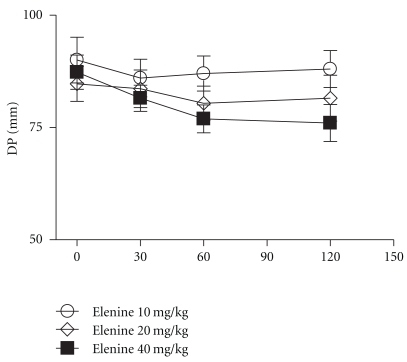
Effect of elenine 10, 20, and 40 mg/kg on Diastolic Pressure (DP mm). The data represent the mean ± SEM for 5 rats at 0, 30, 60, and 120 min.

**Figure 9:**
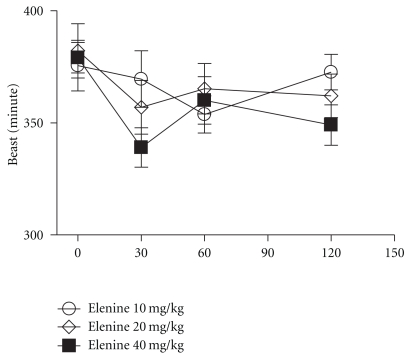
Effect of elenine 10, 20, and 40 mg/kg on heart rate (beats/min). The data represent the mean ± SEM for 5 rats at 0, 30, 60, and 120 min.

The diastolic and systolic pressure of elenine is depicted in Figures 7 and 8, respectively. Elenine at doses of 10 and 20 mg/kg did not cause significant changes in systolic pressure at 30, 60, and 120 minutes respect to basal. Elenine at the dose of 40 mg/kg only caused a slight decrease in the systolic pressure at 60, and 120 minutes. No statistical differences among the three doses of elenine were observed (Figure 7). Elenine at doses of 10, 20, and 40 mg/kg did not cause significant changes in the diastolic pressure at 30, 60, and 120 minutes respect to basal (Figure 8). Moreover, elenine at doses of 10, 20, and 40 mg/kg produced a decrease in the pulse rate (beats/min) at 30, 60, and 120 minutes respect to basal. No statistical differences among the three doses of elenine were observed (Figure 9). 

## 4. Discussion

Elenine showed a moderate toxicity order in mice (254 mg/Kg). No lethality was observed for 7 days following administration of elenine. Besides elenoside, its *β*-D glucoside also showed moderate toxicity (305 mg/Kg) [12]. This finding may explain the use of extracts of *Justicia hyssopifolia* in popular medicine in the Canary Islands [1, 2]. Furthermore, several *Justicia* plant species extracts have been used (*J. prostate, J. simplex, and J. Pectoralis*) in popular medicine [4, 5, 21]. Furthermore, *Justicia pectoralis*, originally from tropical America, is used in folk medicine for treating diseases such as treating pulmonary infections or used as an ingredient in hallucinogenic snuff [21]. 

The effect of elenine was investigated on certain other characteristic actions of general observable behaviour. Thus, nervous activity, observed by the spontaneous movements of mice, gradually decreased 30 min after intraperitoneal injection of elenine. Motor-affective responses in part reflect the effect of elenine on social behaviour. Sensorimotor-responses reflect the capacity of the organism to respond to environmental stimulation. The reduction of motor affective sensorimotor responses indicates an action similar to drugs such as major tranquilizers, sedative hypnotics and narcotics analgesics. Similar results at 25 and 50 mg/Kg of elenoside have been obtained [10]. Muscle tone decreased after 30 min following intraperitoneal injection of elenine and this reduction continued for about 180 min. The loss of righting reflex and hypotonic gait, although slight, was also evident 30 min after administration. The results suggest that the action of elenine is similar to the sedative action of barbiturates and elenoside [10]. 

Hypothermia in mice was observed in a subjective manner (by manual touch) by comparison between the temperature of control animals and animals treated with elenine (10, 20, and 40 mg/kg) to 30, 60, 120, and 180 min. The parameter temperature was measured qualitatively. This is the reason why (+) is used as the score. This manner of scoring using Irwin tests is described by Gerad Vogel [22]. Also, Morales et al. [23] performed the Irwin test on some different extracts of the aerial parts, leaves, bark, and root of plants showing higher activity and a decrease in motor activity, back tonus, reversible palpebral topsis, catalepsy, and strong hypothermia. Moscardo et al. [24] using the Irwin test showed that the reference compounds (chlorpromazine, diazepam and clonidine) induced hypothermia. 

Central nervous activity in rats was examined in the present work using established methods to test the basic function of the CNS. Actions of psychopharmacological agents for example, muscular relaxant activity, locomotor activity and exploratory behaviour pattern were compared with chlorpromazine. In the test concerning the muscular relaxant activity, elenine was found to produce a loss of muscular tone in the traction test in the doses given. Muscular weakness due to elenine was dose dependent and the duration of clinging on the wire net was a few seconds. Elenine and chlorpromazine, 30 min after administration, produced an almost total inhibition of traction performance when a dose of 10, 20, and 40 mg/kg was given. This effect is well known for chlorpromazine [25, 26] and has also been observed with lignans from Justicia pectoralis [21]. These results are in accordance with those obtained by Kuribara et al. [27], so that the test can be used to distinguish between a primary muscular relaxant (diazepam) a primary neuroleptic (chlorpromazine) and a more general central depressant (pentobarbital) activity. Elenine produced a significant decrease in spontaneous locomotor activity in rats when the Varimex test was used. A reduction in the number of counts with two doses of elenine was observed. This effect has also been shown with lignans from Virola elongata bark [28], etoposide [6] and elenoside [13]. 

Varimex and open-field were performed as these are complementary tests. Both tests look at the same parameter. Moreover, the open-field test is used as an animal model of emotional behaviour. Animals with higher levels of motor activity and lower boli rates have been taken to indicate that they exhibit less emotionality, anxiety, fearfulness [29]. Some authors suggest that the open-field test shows aspects of many types of behaviour including exploration, locomotor activity and anxiety [30, 31]. Also, Moscardo showed that reference compounds induced their typical and expected transient effects on neurobehaviour, observed both in the home cage and open-field and on body temperature. Thus, chlorpromazine, diazepam and clonidine induced depressive, anxiolytic or sedative effects associated with hypothermia [24]. 

These results agree with those obtained by the Irwin test, [10, 12] on general behaviour, and a depression of locomotor activity is common to most neuroleptics [25]. Elenine produced a significant decrease in the exploratory behaviour pattern, as can be seen from the results of the open-field test. Reduction of exploratory behaviour (squares and rearing) after treatment with elenine is similar to the actions of many other tranquilizer drugs [32] and elenoside [13]. The increase in the number of boluses and reflex of emotional behaviour in the open-field test could be responsible for the cathartic action, obtained with 10, 20, and 40 mg/kg of elenine, characteristic of elenoside [13] and other antineoplastic compounds [8].

Besides which, because there is a structural analogy between arylnaphthalene lignans and digitalis, cardiac parameters such as blood pressure and heart rate were monitored. Thus, elenine did not cause significant changes in systolic and diastolic pressure at doses of 10, 20, and 40 mg/kg. However, elenine produced a slight decrease in the pulse rate (beats/min). Similar effects have been obtained with cardiac glucosides but of greater amplitude [33]. Moreover, there are no findings with respect to a possible modulation of excitatory/inhibitory neurotransmitter release in the CNS by arylnaphthalene lignans. However, some studies have been reported with other types of lignans. Zhang and Niu [34] have found that schizandrol (*Schizandra *lignan) exerts inhibitory effects on the central nervous system. For the purpose of elucidating the mechanism of inhibition, the concentration of monoamine neurotransmitters and metabolites in the rat brain as well as the effects of schizandrol A on some receptors were determined by the ion-pairing reversed-phase liquid chromatography with electrochemical detection method and comparative binding assay. In the neurotransmitter studies, significant elevations of dopamine and its metabolite (DOPAC) (in striatum) and DA (in hypothalamus) were observed after i.p. administration of 50 mg/kg or 100 mg/kg of schizandrol A, but the receptor binding experiments showed that schizandrol A had no affinity for dopamine D1 and D2 receptors. Serotonin receptors and *α*-1, *α*-2 adrenergic receptors did not affect the binding of dopamine D1 and D2 receptors. These results indicate that the inhibition exerted by schizandrol A on the CNS may be related to the dopamine system and the increase of dopamine turnover has nothing to do with dopamine receptors. Furthermore, *Valerian officinalis* is used in the traditional medicine of many cultures as a mild sedative and tranquilizer or as an aid to induce sleep. The major constituents include monoterpene bornyl acetate, sequiterpene valerenic acid, and other types of sesquiterpene and lignans. Some of these constituents have been shown to have a direct action on the amygdaloid body of the brain, and valerenic acid has been shown to inhibit enzyme-induced breakdown of GABA in the brain resulting in sedation. Another finding of the lignan hydroxyl pinoresinol shows its ability to bind benzodiazepine receptors [35].

Moreover, arylnaphtalene lignans could be involved in several phenomena in the brain of rat, and further studies are necessary to elucidate the mechanism of excitatory/inhibitory neurotransmitters release in the CNS. 

On the basis of the above findings of the present investigation, it can be concluded that elenine has a CNS-depressant action, mostly similar to that of elenoside and other psychopharmacological agents but with lower doses than those used with elenoside. This study supports the central sedative and relaxant properties of elenine and its possible application in anxiety conditions. The sedative properties of this lignan might be relevant to its future therapeutic application. 

## Figures and Tables

**Table 1:** General activity and time-dose response summary showing the average data score.

	Time after drug (min)															
	30				60				120				180			

Dose (mg/Kg)^a^	C	10	20	40	C	10	20	40	C	10	20	40	C	10	20	40
*Behavioural*																
(1) Spontaneous activity																
Body position	4	2.8*	2*	1.8*	4	2.6*	2.8*	1.6*	4	2.2*	1.8*	1.6*	4	2.2*	1.8*	1.6*
Locomotor activity	4	3*	2*	1.8*	4	2*	1.6*	1.5*	4	2*	1.5*	1.5*	4	2*	1.5*	1.5*
(2) Motor-affective response																
Transfer arousal	4	3.1*	2.5*	2.3*	4	2.7*	2.3*	2.2*	4	2.4*	2.2*	2.2*	4	3*	2*	2*
Touch escape	4	3.6*	2.3*	2*	4	3.3*	2.3*	1.8*	4	3*	2*	1.8*	4	3*	2*	1.8*
Positional passivity	0	0	2*	1.9*	4	0	2*	1.9*	4	0	2*	1.9*	4	0	2*	1.9*
(3) Sensorimotor response																
Toe pinch	4	3.2*	2*	2*	4	3*	2*	1.7*	4	3*	2*	1.7*	4	3*	2*	1.7*
Corneal	4	3*	2.5*	2.5	4	3*	2.5*	2.5*	4	3*	2.5*	2.5*	4	3*	2.5*	2.5*
Pinna	4	3.4	3.2*	2.8	4	3.4*	3.2*	2.8*	4	3.4*	3.2*	2.8*	4	3.4*	3.2*	2.8*
(4) Posture																
Tail elevation	2	2	2	2	2	2	2	2	2	2	2	2	2	2	2	2

*Neurologic*																
(1) Muscle tone																
Body tone	4	3.5*	3.*	3*	4	3.2*	2.8*	2.6*	4	3*	2.6*	2.6*	4	3*	2.6*	2.6*
Abdominal tone	4	3.3*	2.6*	2.4*	4	3*	2.6*	2.4*	4	2.8*	2.2*	2*	4	2.8*	2.2*	2*
Limb tone	4	3.6*	3.3*	3*	4	3.6*	3*	3*	4	3.6*	3*	3*	4	3.6*	3*	3*
Grip strength	4	3.4*	2.8*	2.4*	4	3.4*	2.6*	2.4*	4	3.4*	2.6*	2.2*	4	3.4*	2.6*	2.2*
Wire manoeuvre	0	0.5*	0.7*	1.2*	0	0.6*	0.9*	1.2*	0	0.7*	0.9*	1.2*	4	0.7	0.9*	1.2*
(2) Equilibrium and gait																
Righting reflects	0	0.2*	0.3*	0.5*	0	0.5*	0.6*	0.8*	0	0.5*	0.6*	0.80.1*	0	0.5*	0.7*	0.8*
Hypotonic gait	0	0.4*	0.7*	0.8*	0	0.6*	0.8*	0.8*	0	0.6*	0.8*		0	0.6*	0.9*	1*
(3) CNS excitation																
Tremors	0	0	0	0	0	0	0	0	0	0	0	0	0	0	0	0
Twitches	0	0	0	0	0	0	0	0	0	0	0	0	0	0	0	0
Convulsions	0	0	0	0	0	0	0	0	0	0	0	0	0	0	0	0

*Autonomic*																
(1) Eyes: Palpebral closure	0	0	0.4*	0.6*	0	0.4*	0.5*	0.6*	0	0.4*	0.5*	0.6*	0	0.4*	0.5*	0.6*
(2) Secretion Excitation																
Diarrhoea		+	+	+		+	+	+								
(3) General																
Piloerection	0	0	0	0	0	0	0	0	0	0	0	0	0	0	0	0
Hypothermia						+	+	+		+	+	+		+	+	+
Respiratory rate	4	3.8*	3.6*	3.4*	4	3.8*	3.6*	3.4*	4	3.8*	3.6*	3.4*	4	3.8*	3.6*	3.4*
Skin colour	4	4	4	4	4	4	4	4	4	4	4	4	4	4	4	4

(a) C, control, 10, 20, and 40 mg/Kg elenine. *P < .05 versus control group.

**Table 2:** number of animals with loss of spontaneous movement, loss of muscle tone, and loss of righting reflex against the number of observations before and after 1, 2, and 3 h of treatment.

	Loss of spontaneous movement					Loss of muscle tone					Loss of righting reflex				
Treatment^(a)^	C	E-10	E-20	E-40	CHL	C	E-10	E-20	E-40	CHL	C	E-10	E-20	E-40	CHL
**Before**	**0/10**	0/10	0/10	0/10	0/10	**0/10**	0/10	0/10	0/10	0/10	**0/10**	0/10	0/10	0/10	0/10
**1h**	**0/10**	3/10*	6/10*	7/10*	8/10*	**0/10**	3/10*	4/10*	6/10*	7/10*	**0/10**	0/10	1/10	3/10*	3/10*
**2h**	**0/10**	4/10*	7/10*	8/10*	9/10*	**0/10**	4/10*	6/10*	7/10*	8/10*	**0/10**	0/10	1/10	3/10*	3/10*
**3h**	**0/10**	4/10*	7/10*	8/10*	9/10*	**0/10**	6/10*	6/10*	7/10*	8/10*	**0/10**	0/10	1/10	3/10*	3/10*

(a) C, control, 10, 20, and 40 mg/Kg elenine; CHL, chlorpromazine 10 g/Kg. *P < .05 versus control group.
